# Depth-Resolved Distribution of Particle-Attached and Free-Living Bacterial Communities in the Water Column of the New Britain Trench

**DOI:** 10.3389/fmicb.2018.00625

**Published:** 2018-04-04

**Authors:** Rulong Liu, Li Wang, Qianfeng Liu, Zixuan Wang, Zhenzhen Li, Jiasong Fang, Li Zhang, Min Luo

**Affiliations:** ^1^Shanghai Engineering Research Center of Hadal Science and Technology, College of Marine Sciences, Shanghai Ocean University, Shanghai, China; ^2^State Key Laboratory of Marine Geology, Tongji University, Shanghai, China; ^3^State Key Laboratory of Geological Process and Mineral Resources, Department of Earth Sciences, China University of Geosciences, Wuhan, China; ^4^Laboratory for Marine Biology and Biotechnology, Qingdao National Laboratory for Marine Science and Technology, Qingdao, China; ^5^Department of Natural Sciences, Hawaii Pacific University, Honolulu, HI, United States

**Keywords:** particle-attached, free-living, bacteria, New Britain Trench, vertical variation, abundance, diversity

## Abstract

Particle-attached (PA) and free-living (FL) microorganisms play significant but different roles in mineralization of organic matter (OM) in the ocean. Currently, little is known about PA and FL microbial communities in bathyal and abyssal pelagic waters, and understanding of their diversity and distribution in the water column and their interactions with environmental factors in the trench area is limited. We investigated for the first time the variations of abundance and diversities of the PA and FL bacterial communities in the epi-, bathy-, and abyssopelagic zones of the New Britain Trench (NBT). The PA communities showed decreasing species richness but increasing relative abundance with depth, suggesting the increasing ecological significance of the PA bacteria in the deep ocean. The abundance and diversity of PA and FL bacterial communities in the NBT water column appeared to be shaped by different sets of environment factors, which might be related to different micro-niches of the two communities. Analysis on species distribution suggested that the differences between PA and FL bacteria communities mainly resulted from the different relative abundance of the “shared taxa” in the two types of communities. These findings provide valuable information for understanding the relative ecological roles of the PA and FL bacterial communities and their interactions with environmental factors in different pelagic zones along the vertical profile of the NBT water column.

## Introduction

The pelagic deep ocean contains 70% of the ocean’s microbial cells and 60% of its heterotrophic activity, playing a significant role in regulating the biogeochemical cycles of the earth (Arístegui et al., 2009). About 1–40% of the photosynthetically produced organic matter (OM) is exported vertically in the form of sinking particulates (POM), serving as major source of organic matter in the deep ocean (Ducklow et al., 2001; Herndl and Reinthaler, 2013). During sinking, particles are effectively colonized by particle-attached (PA) microorganisms that convert the high-molecular weight organic matter into small substrates available for cellular metabolism (Arnosti, 2011). A fraction of the POM is decomposed and released as dissolved organic matter (DOM) into the surrounding seawater, forming a DOM plume which is further utilized by free-living (FL) microbes (Azam and Malfatti, 2007; Arnosti, 2011; Jiao et al., 2014; Fang et al., 2015). Thus, the fate of OM in the deep ocean is largely determined by the abundance, diversity and activities of the PA and FL microbial communities along the depth profile of the water column (Herndl and Reinthaler, 2013; Jiao et al., 2014; Fang et al., 2015).

The physical and chemical conditions in the water column of the ocean are not uniform, and great variations exist for environmental factors with increasing depth (Jamieson et al., 2010). These factors provide strong selection stresses on microbial communities along the vertical water column. As a result, clear depth stratification of microbial communities has been widely reported in the open ocean (DeLong et al., 2006; Brown et al., 2009; Agogué et al., 2011; Nunoura et al., 2015, 2016; Walsh et al., 2016). However, the majority of the existing studies mainly targeted the whole microbial community. As the PA and FL assemblages play ecologically distinct roles in mediating biogeochemical cycles in the ocean (Azam and Malfatti, 2007; Arnosti, 2011; Jiao et al., 2014; Fang et al., 2015), understanding compositional differences between the two communities as well as their relative responses to the changes of environmental factors is important. Currently, the PA and FL communities have been reported to harbor different groups of microbial lineages in various marine environments including coastal or surface water (Mohit et al., 2014; Zhang et al., 2016), bathypelagic water (Wilkins et al., 2013; Salazar et al., 2016; Milici et al., 2017) or even extreme depths of hadalpelagic habitats (>6000 m) (Eloe et al., 2011b; Tarn et al., 2016). However, these studies mainly focus on the shallower waters or a particular depth in the deep ocean, and understanding on variations of PA and FL communities along the vertical profile is limited (Li et al., 2015; Milici et al., 2017). Existing studies generally revealed different patterns of vertical variations between the PA and FL bacterial communities in the water column above the bathypelagic depth (Li et al., 2015; Milici et al., 2017). It is not known how the abundance and diversity of PA assemblages change relative to those of their FL counterparts from the surface to the abyssopelagic zone. On the other hand, biogeographical distribution of the PA and FL microbial communities at depths around 4000 m in the global ocean was found to be controlled by different sets of environmental factors (Salazar et al., 2016), suggesting that PA and FL microbes may respond differently to environmental conditions at a horizontal scale in bathypelagic waters. It is not clear whether the two microbial communities respond differently to the changes of environmental conditions along vertical scale from surface to abyssopelagic depths, and if so, what are the major factors controlling the vertical variations of PA and FL communities?

The hadal trenches are the deepest part of the ocean. With unique tectonic, topographic, bathymetric and hydrographic features, trenches have been recently discovered as hotspots of microbial activities in the deep ocean (Glud et al., 2013), probably playing an important role in marine biogeochemical cycles (Jamieson et al., 2010; Ichino et al., 2015; Liu et al., 2017). However, studies conducted on PA and FL microbial communities in trench areas are still scarce (Eloe et al., 2011b; Tarn et al., 2016), little is known about the relative changes of PA and FL microbial fractions along the vertical gradients of water column of the trenches.

In this study, we investigate for the first time the changes of abundance and diversity of the PA and FL bacterial communities in the water column of the New Britain Trench (NBT). Water samples were collected along the vertical profile from the surface to the abyssopelagic zone. The objectives are to (1) determine the abundance and diversity of PA and FL bacterial communities from the surface to the abyssopelagic zone; (2) reveal the succession of PA and FL bacterial taxa in response to environmental factors in the water column and the implications for biogeochemical cycles in the oceans.

## Materials and Methods

### Water Sampling and Physical, Geochemical Parameter Measurements

Water samples were taken in August 2016 at station W (06°59.5477′S, 149°46.6129′E) in the western New Britain Trench during a cruise aboard M/V *Zhang Jian*. The full depth of this station is 6700 m, and water samples were collected from 75, 200, 1000, 2000, 3000, 4000, 5000, and 6000 m using Niskin bottles fitted on a Sea-Bird Carousel equipped with a conductivity-temperature-depth (CTD) sensor (Sea-Bird SBE 911plus). For each depth, around 50 L of water was taken and was processed immediately onboard the ship: (1) duplicate 20 L of the water were filtered sequentially through 3 and 0.2 μm polycarbonate filters (PC, Millipore) to collect PA and FL microbes, respectively. Duplicated filters were obtained for each size fraction and were immediately stored at -80°C; (2) for POC and PON analysis, 7–9 L of the water was filtered through 0.7 μm nominal pore size glass fiber filters (GF-75, Whatman) which were pre-combusted in a muffle furnace at 500°C for 12 h. The membrane filters were stored at -20°C after filtration. The filtrates were also stored at -20°C for DOC analysis; (4) Triplicated 100 mL of the water were fixed in paraformaldelhyde (PFA, 1% final concentration) and stored for later microscopic counting of the microbial cells; (5) The remaining water was stored at -20°C for inorganic nutrient measurements.

Physical characteristics of the water including depth, salinity and temperature were measured with CTD sensors during sampling. Concentrations of POC and PON were determined with a PE2400 Series II CHNS/O analyzer (Perkin Elmer, United States) (Chen et al., 2008). Dissolved organic carbon was measured using a Shimadzu TOC-VCHP analyzer with a TNM-1 Total Nitrogen module (Shimadzu Corp., Japan). Seawater nutrients (NO_3_^-^, NO_2_^-^, NH_4_^+^, and PO_4_^3-^) were determined using a QuAAtro autoanalyzer (Seal Analytical, United Kingdom).

### Cell Counting

In laboratory, PFA fixed cells were filtered onto 0.22 μm pore size black polycarbonate filters (diameter, 25 mm; Whatman-Nucleopore) and stained with DAPI (4′,6-diamidino-2-phenylindole). Bacterial cells were counted under epifluorescent microscopy (Nikon microscope, model Eclipse Ni-U). For each sample, no less than 20 microscopic fields were counted and cell abundance was calculated according to Liu et al. (2010).

### DNA Extraction and PCR Amplification

In this study, we applied two different DNA extraction methods and two primer sets targeting different hypervariable regions of 16s rRNA gene. Two sets of data were generated for each of the samples, and were then combined into one data set according to the method described in a later section about sequences analysis.

For each depth, duplicate PC filters were obtained for both 3 and 0.2 μm fractions (as described above). The 1st filter was lysed using lysozyme (30 mg ml^-1^) and then extracted with AllPrep DNA/RNA Mini Kit (Qiagen). The 2nd filter was extracted with FastDNA^TM^ SPIN Kit for Soil DNA Extraction (MP) according to manufacturer’s protocols. The concentration and quality of final DNAs were determined by NanoDrop 2000 UV-vis spectrophotometer (Thermo Scientific, Wilmington, DE, United States). DNA samples from the 1st replicates were amplified with barcoded primers 338F (5′-ACTCCTACGGGAGGCAGCAG-3′) and 806R (5′-GGACTACHVGGGTWTCTAAT-3′) targeting the V3–V4 hypervariable regions. DNAs from the 2nd replicates were amplified with barcoded primers 515F (5′-GTGCCAGCMGCCGCGG-3′) and 907R (5′-CCGTCAATTCMTTTRAGTTT-3′) targeting the V4–V5 hypervariable regions of 16s rRNA gene. PCR reactions were conducted using a thermocycler PCR system (GeneAmp 9700, ABI, United States) with the following program: 5 min of denaturation at 95°C, 27 cycles of 60 s at 95°C, 60 s at 55°C for annealing, and 60 s at 72°C for extension, and a final extension step at 72°C for 10 min. PCR reactions were performed in triplicate 50 μL mixture with each containing 1X ExTaq buffer, 0.2 mM of each dNTP, 0.2 μM of each primer, 1 U of ExTaq polymerase (TAKARA, China) and 1 μl DNA. PCR products were purified from 2% agarose gel using AxyPrep DNA Gel Extraction Kit (Axygen Biosciences, Union City, CA, United States) and quantified using QuantiFluor^TM^-ST (Promega, United States). DNAs from PA and FL fractions of 3000 m and PA fraction of 5000 m were failed in PCR amplification with 338F/806R, so these samples were excluded from the diversity analysis.

### llumina MiSeq Sequencing and Sequences Processing

Purified amplicons were pooled in equimolar and paired-end sequenced (2 × 300) on an Illumina MiSeq platform (Illumina, San Diego, CA, United States) according to standard protocols by Majorbio Bio-Pharm Technology Co. Ltd. (Shanghai, China). Raw fastq files were demultiplexed, quality-filtered by Trimmomatic (Bolger et al., 2014) and assembled by FLASH (Magoc and Salzberg, 2011) with the following criteria: (i) Reads with an average quality score < 20 over a 50 bp sliding window at any site were truncated; (ii) Primers were checked by allowing 2 nucleotides mismatching, and reads containing ambiguous bases were removed; (iii) Sequences with overlaps of more than 10 bp were assembled according to their overlap sequence. Chimeric sequences were identified and removed using UCHIME and operational taxonomic units (OTUs) were clustered with 97% similarity cutoff using UPARSE (version 7.1^1^. The taxonomy of each OTU was assigned by RDP Classifier^2^ against the Silva16S rRNA database (SSU123) with a confidence threshold of 70%.

### Diversity Analysis and Statistical Tests

Two datasets, named “Qiagen-338-806” and “MP-515-907” were generated for each of the samples, and were combined into one dataset according to Vahjen et al. (2010). Briefly, the sequences from two datasets were randomly resampled to ensure each sample from the two datasets has the same number of sequences (Ghiglione et al., 2012). This step resulted in two new sets of data with 20,118 sequences for each of the samples. These two datasets were then merged by taking only sequences from the primer set that yielded the higher number of reads for a specific OTU assignment in a sample (Vahjen et al., 2010), which generated a new dataset with 35,877 to 40,046 sequences for the 13 samples tested. The newly generated datasets showed much improved resolution of microbial diversity (see Results).

The diversity indices including species richness (at OTUs level), Pielou’s evenness, Shannon index, Simpson index were calculated with PRIMER 6.0 package using OTU table of the merged data set generated as described above. Venn diagram was produced with VENNY 2.1 (Oliveros, 2007–2015) to show the changes of OTU distribution in PA and FL fractions at different depth. Lists of OTUs that were exclusively present in either PA or FL fractions and those present in both fractions were exported from Venn analysis, and the relative abundance of those listed OTUs in the total sequences of a particular sample were manually examined in the OTU table. A Bray-Curtis similarity matrix was constructed based on species composition at OTU level to show the β-diversity between different samples and was visualized using non-metric multidimensional scaling (nMDS) and hierarchical clustering (UPGMA) using the PRIMER 6.0 package. One way Analysis of Similarity (ANOSIM) was performed to test for differences between different groups. Samples that were clustered together in nMDS were regarded as the same group and used to compare with other groups. Similarity percentage (SIMPER) (Clarke and Warwick, 2001) was used to identify major OTUs responsible for dissimilarity between different bacterial groups. Relationships between the species composition of bacterial communities and environmental parameters were assessed using canonical correspondence analysis (CCA) (CANOCO version 4.5) (Ter Braak, 1986). The environmental factors we tested in this study include physical parameters (pressure, temperature, and salinity), inorganic nutrients (nitrate, nitrite, ammonia, phosphate) and organic contents (POC, PON, and DOC). All of the environmental factors were included in the stepwise “forward selection” with Monte Carlo permutation test (999 permutations) to produce an optimal CCA model, and environmental factors with significant conditional effects in either PA or FL bacterial community were selected.

### Quantitative PCR Determining the Copy Numbers of Bacterial 16s rRNA Gene

Each DNA sample was tested with SYBR Green^®^ qPCR assay Eub338F/Eub518R (Thijs et al., 2017) to determine the copy number of PA and FL bacterial 16s rRNA gene. All qPCR assays were performed in 20-μl reactions, each contained 2 μl of DNA and 500 nM of each primer in 2X AceQ SYBR Green^®^ qPCR Master Mix (Vazyme). Duplicate reactions were used for each sample. The thermal cycles on the ABI 7500 (Applied Biosystems) were the following: initial denaturation (95°C) for 5 min, followed by 40 cycles of denaturation (95°C) for 5 s, annealing at 55°C for 30 s and extension at 72°C for 40 s. A melting curve analysis was included in each run of qPCR to check the specificity of amplification. Cycle of threshold (Ct) was calculated using the ABI 7500 software with the auto-baseline and auto-threshold functions. Standard curves of Ct vs. gene copy numbers were constructed using serial dilutions of linearized plasmids containing amplicon fragment of Eub338F/Eub518R.

## Results

### Physical-Chemical Parameters and Microbial Cell Abundance

**FIGURE 1:**
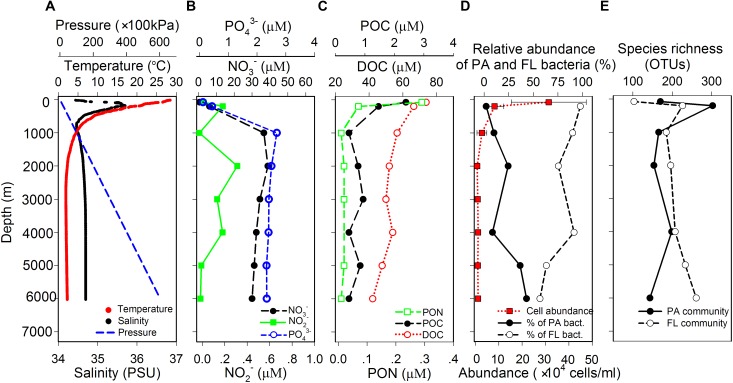
Vertical variations of **(A)** physical parameters, **(B)** concentration of inorganic nutrients, **(C)** concentration of organic matter, **(D)** cell abundance of total prokaryotes, relative abundance of the PA and FL bacteria, and **(E)** species richness of the PA and FL bacterial communities in the water column of the New Britain Trench.

Temperature continuously decreased from 28.4°C at surface water to around 2.2°C at 2000 m and maintained at around 1.9–2.3°C from 2000 to 6000 m. Salinity showed gradual increase from 34.4 PSU at surface to 35.7 PSU at 180 m, decreased to 34.5 PSU at 1000 m, and then maintained at around 34.5–34.7 PSU until 6000 m (**Figure 1A**). Nitrate and phosphate concentrations were the lowest at surface water and the highest at around 1000 m, while the concentration of nitrite fluctuated along the whole water column with a decreasing trend (**Figure 1B**). POC and PON concentrations were continuously decreasing with increasing depth. DOC showed similar trend, but its concentration remained relatively constant below 1000 m (**Figure 1C**). Microbial cell abundance ranged from 3.14 ± 1.61 × 10^5^ cells/ml in surface water to 7.17 ± 9.23 × 10^3^ cells/ml at 2000 m and remained relatively constant from 2000 to 6000 m with an average value of 9.75 × 10^3^ cells/ml (**Figure 1D**). It is interesting to note that the relative abundances of PA and FL bacteria, as determined by qPCR quantification of bacterial 16s rRNA genes, showed reversed vertical trends. The relative abundance of PA bacteria increased and that of FL bacteria decreased with depth (**Figure 1D**).

### Vertical Variations in Diversity of the PA and FL Bacterial Communities

It has been widely reported that different DNA extraction methods and primer sets targeting different hypervariable regions of 16s rRNA gene would reveal distinct species compositions of natural microbial communities (Vahjen et al., 2010; Cai et al., 2013; Starke et al., 2014; Wang et al., 2015). In some extreme cases, dominant taxa revealed from one combination of DNA extraction and primer set can become rare taxa or even disappear in another combination (Cai et al., 2013; Starke et al., 2014). Therefore, combining data from different primer sets has been suggested to maximize the detection of microbial diversity (Vahjen et al., 2010; Wang et al., 2015). In this study, we applied two different DNA extraction methods and two primer sets targeting different hypervariable regions of 16s rRNA gene. Two sets of data were generated for each of the samples, and were then combined into one dataset. Similar to previous findings (Vahjen et al., 2010; Cai et al., 2013; Starke et al., 2014; Wang et al., 2015), the species composition of the microbial communities showed great variations between the two original datasets (Supplementary Figures S1, S2). In addition, the original datasets “Qiagen-338-806” and “MP-515-907” revealed 2,438 and 1,947 OTUs, respectively. The combined dataset had 3,313 OTUs (Supplementary Table S1), suggesting greatly improved resolution of microbial diversity.

Species richness of the PA and FL bacterial communities showed distinct vertical trends: richness for the PA assemblages were the highest at 200 m and showed a general trend of decreasing with depth; whereas richness for the FL assemblage increased with depth, with the highest value at 6000 m (**Figure 1E**). Different from species richness, changes of Pielou’s evenness and Shannon and Simpson indexes showed similar patterns of vertical variation for both PA and FL bacterial communities, with lowest value at 1000 m and highest values at 200 and 6000 m (Supplementary Figure S3).

**FIGURE 2:**
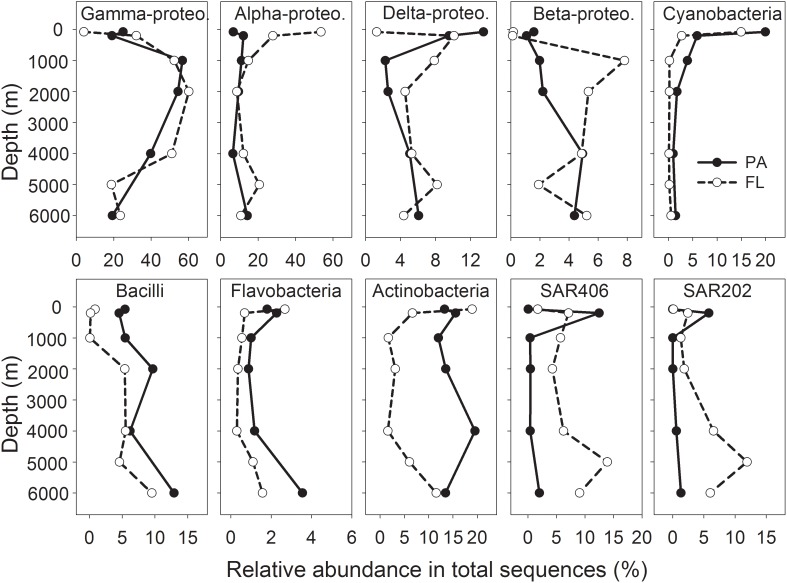
Vertical variations of major bacterial taxa at the class level for PA and FL bacterial communities. Shown are the top 10 classes that have the highest average abundances among all samples.

Proteobacteria was the single dominant phylum in the entire water column of the NBT, accounting for 41.8–82.7% of the total sequences retrieved from different depths, with relatively higher proportions at 1,000 – 4,000 m depth and lower proportions at surface and abyssal depth (**Figure 2** and Supplementary Figure S4). Gamma-, Alpha-, Delta-, and Beta-proteobacteria were the dominant classes of Proteobacteria, without clear differences between the two lifestyles (PA vs. FL) (**Figure 2** and Supplementary Figure S4). Cyanobacteria were most abundant at 75 m in both the PA and FL bacterial communities, but their abundance declined sharply with depth, and PA maintained relatively higher abundance than FL communities at all depths (**Figure 2**). The relative abundance of Bacili class gradually increased with depth and was constantly higher in the PA than the FL communities in the whole water column (**Figure 2**). The relative abundances of Actinobacteria and Flavobacteria were consistently higher in the PA than in the FL bacterial communities in waters deeper than 200 m (**Figure 2**). In contrast, the SAR406 and SAR 202 clades displayed much higher relative abundances in the FL than in the PA communities in deep waters (**Figure 2**).

**FIGURE 3:**
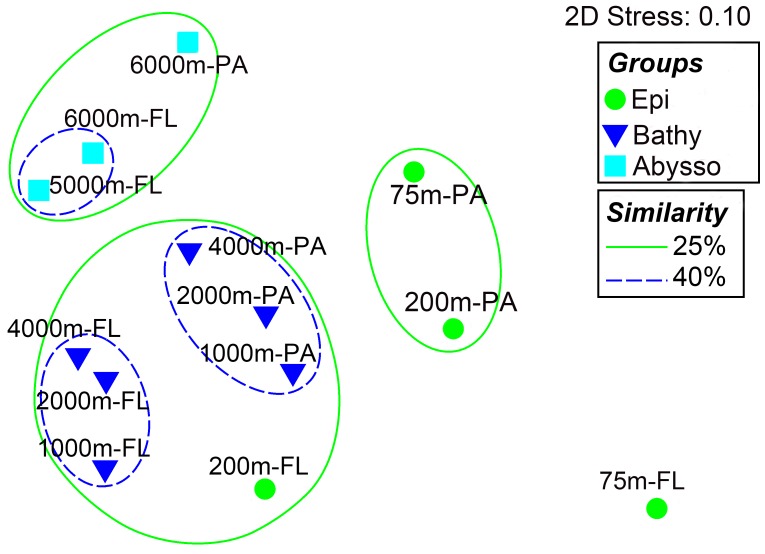
Non-parametric multi-dimensional scaling (nMDS) based on Bray-Curtis similarity matrix of species composition at the OTU level. Different symbols represent different pelagic zones the samples were taken from. Circles with solid lines indicate the similarity level of 25% while circles with dotted lines indicate the 40% similarity.

Non-metric multidimensional scaling (nMDS) and hierarchical clustering (UPGMA) analyses showed obvious differences in species composition between the communities (**Figure 3**). Overall, the samples were clustered into three large groups, roughly representing different pelagic zones along the depth profile, i.e., the epipelagic zone (75 and 200 m), the bathypelagic zone (1000, 2000, and 4000 m), and the abyssopelagic zone (5000 and 6000 m). It was also clear that, within each pelagic zone, the respective PA and FL bacterial communities tend to form different groups (**Figure 3**). Analysis of similarity (ANOSIM) supported the nMDS groupings: the differences between the pelagic zones were significant (Global *R* = 0.708, *P* = 0.001, **Table 1**), while the differences between the PA and FL fractions in the whole water column were much lower (*R* = 0.187, *P* = 0.065, **Table 1**), suggesting that vertical depth stratification is more important than trophic lifestyle in shaping the species composition of bacterial communities in the water column of the NBT.

**Table 1:** Analysis of similarity (ANOSIM) between trophic lifestyles (PA vs. FL) (Comparison 1) and between the pelagic zones (Comparison 2).

ANOSIM	*R*	*P*
**Comparison 1**		
PA vs. FL	0.187	0.065
**Comparison 2**		
Epi vs. Bathy	0.667	0.005
Epi vs. Abysso	0.741	0.029
Bathy vs. Abysso	0.840	0.012
Global	0.708	0.001


*The *R-*value shows differences between the two groups compared, while the *P-*value indicates the level of significance.*

**FIGURE 4:**
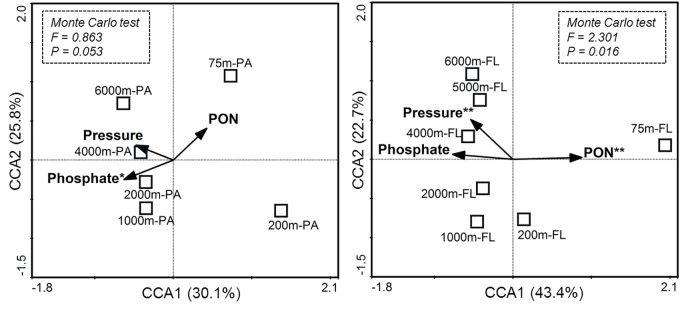
Ordination diagram of the first two axes in canonical correspondence analysis (CCA) for particle-attached (PA) and free-living (FL) bacterial communities from different depths. Each square represents an individual sample. The vectors indicate constrained environmental variables that showed significant effects on species composition of either PA or FL bacterial community, as assessed by the marginal effect of the terms. For each diagram, environmental variables marked with asterisks are statistically significant (^∗^*P* < 0.05; ^∗∗^*P* < 0.01). The significance of the first canonical axis was tested via Monte Carlo test with 999 permutations, and the results are shown in the rectangles with dotted lines.

Canonical correspondence analysis was further conducted to show the correlations between the major environmental factors and the observed differences in species composition between the PA and FL bacterial communities (**Figure 4**). The results showed that the first two CCA axes explained 55.9% of the variations for species composition of the PA communities, and 66.1% of the variations for the FL communities. Monte Carlo test on significance of the first CCA axes showed that only the CCA ax for FL communities was statistically significant (*P* = 0.016) while that for the PA communities was not (*P* = 0.053). Among the tested environmental factors, phosphate was the statistically significant variable associated with the vertical variations of the PA bacterial communities (*P* = 0.031), while pressure (*P* = 0.008) and PON concentration (*P* = 0.009) were the statistically significant variables associated with the vertical pattern of FL bacterial communities.

### Distribution of Different Bacterial Lineages in PA and FL Fractions

**FIGURE 5:**
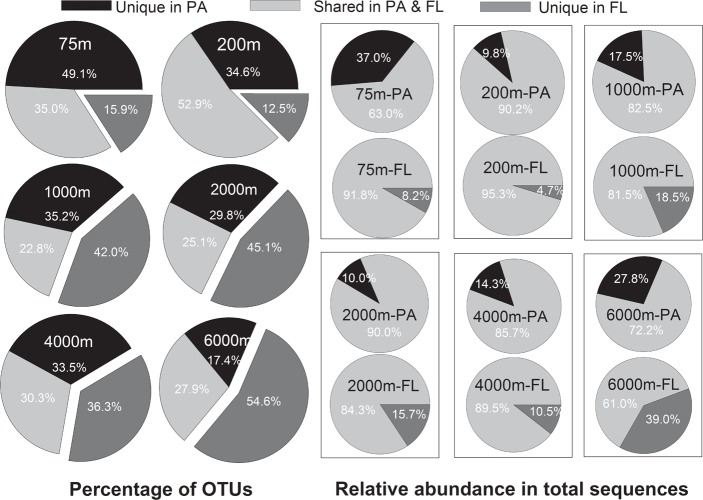
Operational taxonomic units (OTUs) distribution in PA and FL bacterial communities. Larger pie charts at left side show the percentage of shared or unique OTUs in PA and FL bacterial communities at different depths. The smaller pie charts on the right side show the relative abundance of shared or unique OTUs in the total sequences of individual samples at different depths.

Venn analysis showed that the proportion of the shared OTUs between the PA and FL bacterial communities ranged between 22.8% (at 1000 m) to 52.9% (at 200 m), with an average of 32.3%; while the proportion of OTUs present exclusively in the PA communities ranged between 17.4% at 6000 m and 49.1% at 75 m (average value of 33.3%); and that of OTUs present exclusively in FL communities ranged between 15.9% at 75 m and 54.6% at 6000 m, with an average of 34.9% (**Figure 5**). However, when taking into consideration of the relative abundance of each OTU in total sequence reads of different samples, we found that OTUs commonly present in both the PA and FL communities accounted for 61.0–95.3% of the total reads of the samples, with an average of 82.2% (**Figure 5**). In other words, on average 82.2% of the reads from the tested samples belonged to OTUs that present in both the PA and FL bacterial communities.

**FIGURE 6:**
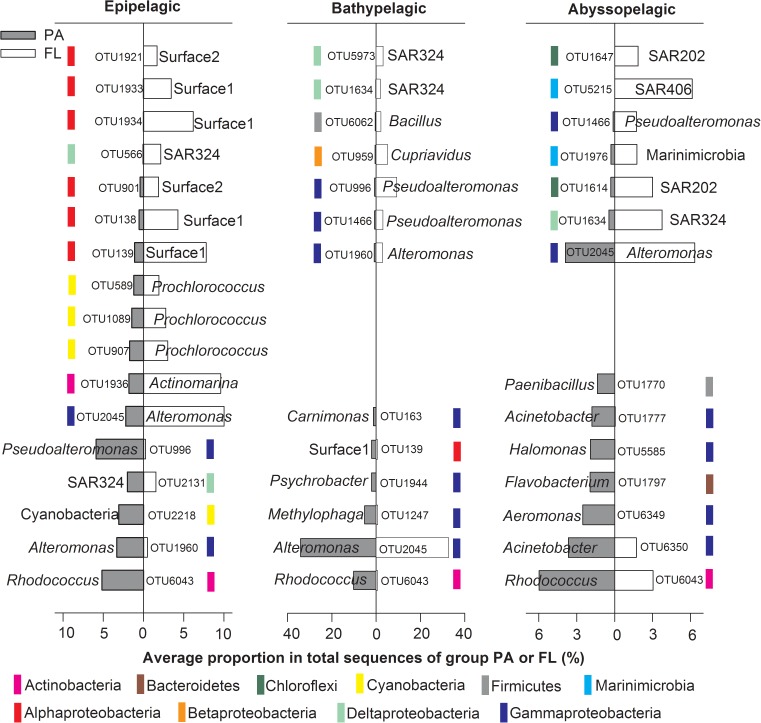
The identified “indicative OTUs” for PA or FL lifestyles at different pelagic zones and their uneven distribution in the two bacterial communities. The “indicative OTUs” were defined as OTUs that contributed ≥ 1% of dissimilarities between PA and FL communities at each pelagic zone, based on the SIMPER analysis. For each OTU, the OTU name and the name of the lowest recognized classification was labeled. Colored bars show the class or phylum the OTUs belong to.

SIMPER analysis was applied to identify the “indicative OTUs” that were responsible for the observed dissimilarities between the PA and FL communities in the NBT water column. In this study, “indicative OTUs” were defined as OTUs that contributed more than 1% dissimilarities between the two bacterial communities (**Figure 6**). As a result, 17, 13, and 14 indicative OTUs were identified from the epipelagic, bathypelagic and abyssopelagic zones, respectively. The OTUs had uneven distributions in the PA and FL bacterial communities, but most of them presented in both types of the bacterial communities. For instance, among the identified indicative OTUs, 16 from the epipelagic zone, 11 from the bathypelagic zone, and 9 from the abyssopelagic zone were present in both the PA and FL bacterial communities; only minor proportions of indicative OTUs were present exclusively in either the PA or FL communities (**Figure 6** and Supplementary Tables S2–S4). OTU6043, an unclassified *Rhodococcus* was abundant in PA communities throughout the whole water column, with much higher relative abundance in PA (5.2–10.2%) than FL (0.0–3.0%) communities (**Figure 6** and Supplementary Tables S2–S4). OTU2045 from *Alteromonas* was the dominant taxon in both PA (2.2–34.1%) and FL (6.3–32.8%) communities throughout the water column, and their preference of lifestyle varied at different depth (**Figure 6** and Supplementary Tables S2–S4). Other indicative OTUs were enriched in either PA or FL communities only in one of the pelagic zones (**Figure 6**).

## Discussion

### Variations in Diversity and Abundance of the PA and FL Bacterial Communities in the NBT Water Column

In this study, the relative abundance of PA bacteria showed an increasing trend with depth (**Figure 1D**), indicating the potentially increasing ecological significance of PA bacterial communities in the deep ocean comparing to shallower depths. This result could also partially explain the accumulating findings in the literature that bacterial communities in the deep ocean are typically enriched for taxa and genomic features with a particle- or surface-associated lifestyle (DeLong et al., 2006; Lauro et al., 2009; Eloe et al., 2011a; Herndl and Reinthaler, 2013; Giovannoni et al., 2014; Thrash et al., 2014). In contrast to the changes of bacterial abundances, species richness of the PA bacterial community showed a general trend of decreasing while that for the FL community increased with depth (**Figure 1E**). As a result, species richness was higher in PA than FL bacterial communities in the surface water (epipelagic zone), and higher in FL than PA communities at the bathyal and abyssal depths (**Figure 1E**). Our results for the epipelagic water are consistent with previous studies conducted at similar depths (Crespo et al., 2013; Bižic-Ionescu et al., 2014; Rieck et al., 2015). For deeper waters, although higher richness in the PA bacterial communities have been reported for samples from the Southern Ocean (Milici et al., 2017) and the Puerto Rico Trench (Eloe et al., 2011b), results from other studies with larger datasets and from wider range of world oceans support our observations, i.e., species richness is higher for FL than PA in the deep ocean (Salazar et al., 2015, global scale bathyal waters; Tarn et al., 2016, multiple sites along the Mariana Trench; Ghiglione et al., 2007, the Mediterranean Sea).

The opposite trends of changes in abundance and species richness may be related to the changes of quantity and bio-availability of organic matter in the particles and the surrounding water along the depth profile and the biogeochemical processes in which the PA and FL communities are involved. The amount and lability of dissolved organic matter decreases with depth in the ocean (Jiao et al., 2010, 2014), leading to the gradual decreasing of heterotrophic bacterial biomass. However, as the descending particles contain higher amount of carbon and nutrients relative to the surrounding water and are preferred hotspots for microbial colonization (Fang et al., 2015; Dang and Lovell, 2016), the rate of decreasing in bacterial biomass on the particles may be less than that in the surrounding waters. As a result, the relative abundance of PA bacteria increased with depth. For species richness, it has been hypothesized that given the constrained space and limited resources on particles, only bacterial taxa that could further hydrolyze the less labile OM could actively colonize on particles in the deeper water. The growth of these taxa may compete with or even inhibit the growth of other attached bacterial taxa (Dang and Lovell, 2016), thus resulting in detachment and reduced species richness of the PA bacteria and increased species numbers of the FL communities in the deep (Kiørboe et al., 2003; Tamburini et al., 2013). Furthermore, PA bacterial decomposition of POM may release labile DOM into the surrounding waters which, because of chemotaxis, would be another possible reason for higher species richness of the FL fractions. In certain cases, chemolithotrophic species can be important source of FL bacteria in the deep ocean (Herndl and Reinthaler, 2013).

### The Effect of Depth and Other Environmental Factors

Beta diversity analysis showed that bacterial communities in the NBT water column mainly clustered according to the pelagic zones. However, the PA and FL communities within each zone tend to grouped separately (**Figure 3**). ANOSIM analysis showed that there were significant differences in microbial communities between the pelagic zones and no significant differences between communities of the two trophic lifestyles (i.e., PA and FL) in the whole water column (**Table 1**). These results suggest that vertical stratification is a key factor in shaping bacterial community structure in the NBT water column.

It is also clear that depth and other environmental factors seem to have different impact on the PA and FL bacterial communities. As shown in CCA analysis, most of the variations in species composition of the FL bacterial communities can be explained by the first two CCA axes (66.1%), but much less so for the PA bacterial communities (55.9%) (**Figure 4**). This finding suggests that the FL bacterial communities are likely more responsive to the depth-related variations of environmental factors tested in this study. Consequently, the magnitude of changes in bacterial diversity with depth was much larger for the FL communities than that for the PA communities (Supplementary Figure S5). Further analysis showed that phosphate exhibited statistically significant correlation with the vertical variations of the PA bacterial communities, and pressure and PON concentration co-varied with the vertical pattern of the FL bacterial communities. The results suggest that the PA and FL bacterial communities may respond to, and be controlled by, different environmental factors. It is reasonably assumed that particles are enriched with organic matter and inorganic matters (e.g., phosphate), and therefore a close co-variation between bacterial communities and phosphate concentration is not unexpected. Previous studies have shown that surface attachment and the subsequent production of shielding biofilm matrix and stress response products may protect bacteria from environmental variability, such as temperature extremes (Wiebe et al., 1992; Dang and Lovell, 2016). Also, the content of organic matter on particles is much higher than that in the bulk seawater (Fang et al., 2015; Dang and Lovell, 2016). Thus, the PA communities may be less affected by variations in the amount of organic matter and physical variables in seawater, comparing to the FL bacterial communities. In contrast, the FL bacteria living in the bulk seawater exposes directly to the effect of physical factors such as pressure, and their survival mainly rely on consumption of DOM (Azam and Malfatti, 2007; Arnosti, 2011; Fang et al., 2015), it is therefore reasonable to observe significant correlations between POM (PON in this study) and physical factors (e.g., pressure) and the vertical variations of FL bacterial community. Pressure has long been recognized as an important factor affecting species composition of marine bacterial communities in laboratory incubation experiments (Grossart and Gust, 2009; Tamburini et al., 2013; Marietou and Bartlett, 2014). However, reports on relationships between pressure and variations of bacterial communities in natural oceanic habitats are scarce. Here, we provide direct evidence showing the significant effect of pressure in shaping bacterial community structure from the surface ocean to the abyssopelagic zone of the New Britain Trench (**Figure 4**), further supporting the potential significance of pressure-adapted piezophilic bacteria and their role in mediating biogeochemical cycles in the deep ocean (Fang et al., 2010, 2015).

### Distribution of Different Bacterial Lineages in PA and FL Fractions at Different Depths

Significant differences in species composition between the PA and FL bacterial communities are frequently reported (Wilkins et al., 2013; Salazar et al., 2016; Milici et al., 2017). It seems reasonable to postulate that many taxa that adapted exclusively to either PA or FL lifestyle exist in the ocean, leading to the overall community level differences. However, our results revealed that, although there were on average 33 and 35% of the total OTUs exclusively presented in the PA and FL communities, respectively, these OTUs only accounted for minor proportions of the total sequences retrieved (**Figure 5**). The majority of the sequences (61.0–95.3%) belonged to OTUs that were shared between the PA and FL communities (**Figure 5**). We further analyzed the major OTUs responsible for discriminating the PA and FL bacterial communities (the “indicative OTUs”). Our results suggest that the majority of the “indicative OTUs” are those that were shared between the PA and FL communities and that had much higher relative abundances in one type of the communities (**Figure 6**). In other words, the differences in community structure between the PA and FL bacterial communities tested in this study are mainly due to the uneven distribution of the shared OTUs in the PA and FL fractions. High percentage of sequences belonging to shared OTUs in PA and FL bacteria have also been observed in several previous studies in surface water (Crespo et al., 2013; Yung et al., 2016) and mesopelagic water (Hollibaugh et al., 2000; Moeseneder et al., 2001; Ghiglione et al., 2007). Our study is the first to extend such observations into bathy- and abyssopelagic waters, and the findings suggest that shared OTUs between PA and FL bacterial communities may be common at different depths and in different locations of the ocean. It is not yet known the exact reasons for the shared OTUs in the PA and FL bacterial communities. It is possible that certain taxa have attained the mechanisms to switch between the two lifestyles for the purpose of accessing carbon and nutrients (Fang et al., 2015; Dang and Lovell, 2016). Alternatively, these OTUs contain sub-OTUs that show sub-OTU level or strain level habitat specialization (Hunt et al., 2008; Yung et al., 2015). Further studies exploring the true reasons behind the shared OTUs in both lifestyles are important for understanding the mechanisms of remineralization of POM and DOM in the ocean.

Although the majority of the sequences belonging to OTUs present in both PA and FL communities, most of these shared OTUs showed obvious enrichment on one lifestyle (**Figure 6**). The enrichment of different bacterial taxa on PA or FL lifestyle was more obvious at the class level: the PA community contained higher proportions of species belonging to Cyanobacteria, Bacilli (Firmicutes), Flavobacteria (Bacteroidetes) and Actinobacteria throughout the water column (**Figure 2**). Due to the phototrophic nature of Cyanobacteria, these bacteria may not be playing an active role in the deep water, but the presence of Cyanobacteria in the PA bacterial communities throughout the water column may serve as an indication of bacteria attachment to and vertical transport of particles and the attached bacteria. It is also possible that this represents an artifact of filtration as the size of certain Cyanobacteria taxa is much greater than the pore size of the filter we utilized (3 μm). Alternatively, the detected Cyanobacteria might be dead cells and serve as substrates for PA bacteria, which are transported to the deep water. Association of taxa from Firmicutes and Bacteroidetes with PA lifestyle has been reported in the bathypelagic oceans globally (Salazar et al., 2015), which could be related to their capability of degrading complex organic matters such as polymers (Cottrell and Kirchman, 2000). Actinobacteria has also been demonstrated to produce various extracellular hydrolytic enzymes and degrade a wide range of organics, including refractory organics such as polycyclic aromatic hydrocarbons and polysaccharides (Chen et al., 2016). Different from their PA counterparts, the FL bacterial communities contained much higher proportions of SAR406 and SAR202, especially in the deep water (**Figure 2**). Enrichment of these two groups in the FL fraction of the deep ocean has also been reported in previous studies (Salazar et al., 2015; Milici et al., 2017). Bacteria from those two groups generally have streamlined genomes and adapted to the oligotrophic conditions in the deep (DeLong et al., 2006; Eloe et al., 2011a; Giovannoni et al., 2014). In addition, the most dominant classes, i.e., Gamma-, Alpha-, and Deltaproteobacteria showed mixed distributions between the PA and FL communities, as reported in the literature (Eloe et al., 2011b; Li et al., 2015; Salazar et al., 2015; Milici et al., 2017). Our results support the hypothesis that high bacterial taxonomic ranks (Class/Phylum) have conserved lifestyles, either in association with particles or as FL (Eloe et al., 2011b; Salazar et al., 2015).

## Conclusion

We investigated for the first time the vertical variations of abundance and diversity of PA and FL bacterial communities from surface ocean to abyssopelagic waters in the New Britain Trench. Our results revealed distinct patterns of PA and FL bacterial communities on their vertical variations of relative abundance, species richness, species compositions as well as their relationships with environmental factors. The PA bacteria showed increasing relative abundance with depth, suggesting the increased ecological significance of the PA lifestyle in the deep ocean. Vertical variations of the species compositions of PA and FL bacterial communities were found to be significantly associated with different sets of environmental factors, and FL bacteria were more responsive to changes of environmental variables tested. The differences on their relationships with environmental factors might be due to the different micro-niches of the PA and FL bacterial communities. Analysis of species distribution between PA and FL communities revealed that majority of sequences are belonging to OTUs shared between two communities, and the enrichment of these shared OTUs in either PA or FL lifestyle was the major reason for differences between the two bacterial communities. These findings provide new insights to the bacterial adaptation of different lifestyles. However, the reasons of shared taxa in both lifestyles and the implications to the marine carbon cycles need to be further addressed in the following studies.

## Data Accessibility

All raw sequences used in this study are publicly available at the NCBI Sequence Read Archive (SRA, http://www.ncbi.nlm.nih.gov/Traces/sra) under accession ID SRP125714.

## Author Contributions

RL, LW, and JF designed the expedition and sampling scheme, analyzed the data, and wrote the article. QL and RL prepared the samples on board. LW, QL, ZW, and ZL conducted the experimental procedures including cell counting, DNA extraction, PCR amplification, qPCR analysis, and bioinformatics analysis. ML measured all chemical parameters. RL, LW, QL, ZW, ZL, JF, and LZ made comments and suggestions to improve the manuscript.

## Conflict of Interest Statement

The authors declare that the research was conducted in the absence of any commercial or financial relationships that could be construed as a potential conflict of interest.

## Footnotes

1 http://drive5.com/uparse/

2 http://rdp.cme.msu.edu/
