# Supplementary material for: Depth-Resolved Distribution of Particle-Attached and Free-Living Bacterial Communities in the Water Column of the New Britain Trench
Source: Front Microbiol. 2018 Apr 4;9:625. doi: 10.3389/fmicb.2018.00625 (PMC5893722; doi:10.3389/fmicb.2018.00625)
Supplement: Supplementary file 1 [file Data_Sheet_1.DOCX]

Supplementary Material

**Depth-resolved Distribution of Particle-attached and Free-living Bacterial Communities in the Water Column of the New Britain Trench**

**Rulong Liu^1†^, Li Wang^1†^, Qianfeng Liu^2^, Zixuan Wang^1^, Zhenzhen Li^3^, Jiasong Fang^1,4,5*^, Li Zhang^3^, Min Luo^1^**

***Correspondence:**

Jiasong Fang

[jsfang@shou.edu.cn](mailto:jsfang@shou.edu.cn)

†, These authors contributed equally to this work and are co-first authors

# Supplementary Figures and Tables

## Supplementary Figure

**
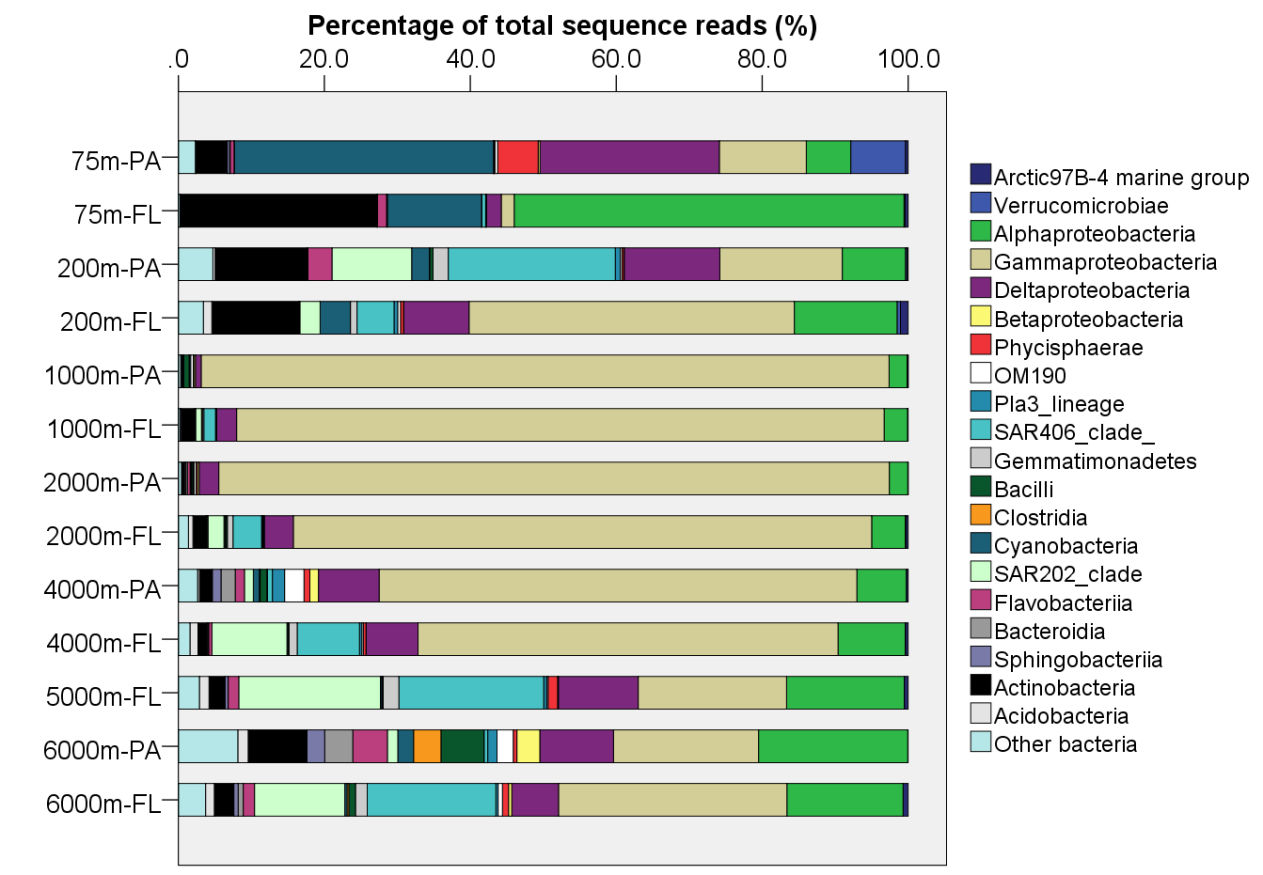
**

**Fig. S1.** The composition of bacterial communities at the class level. The analysis was based on the dataset “Qiagen-338-806”.

**
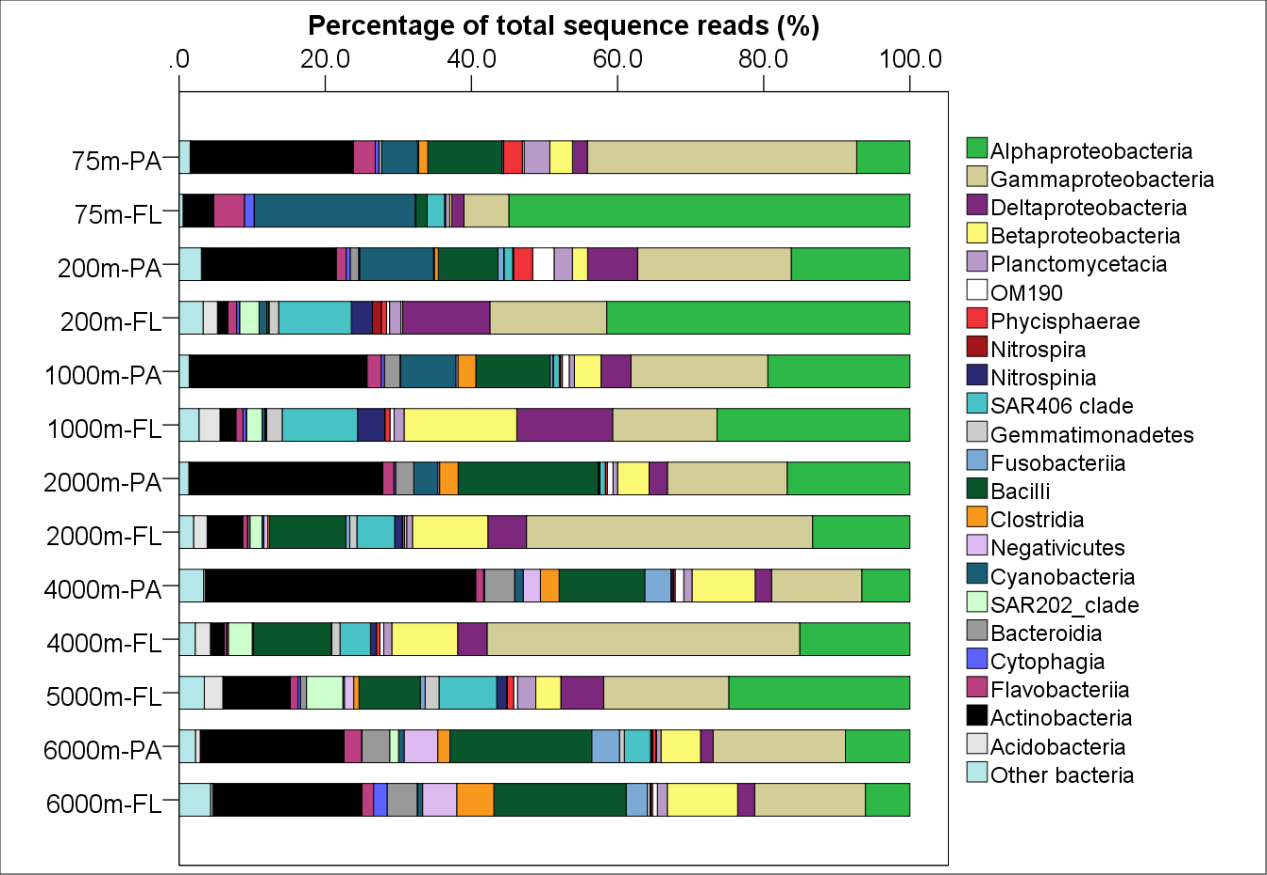
**

**Fig. S2.** The composition of bacterial communities at the class level. The analysis was based on the dataset “MP-515-907”.

**Fig. S3** Changes of species richness, Pielou’s evenness, Shannon and Simpson indexes of the PA and FL bacterial communities along the water column of the New Britain Trench.


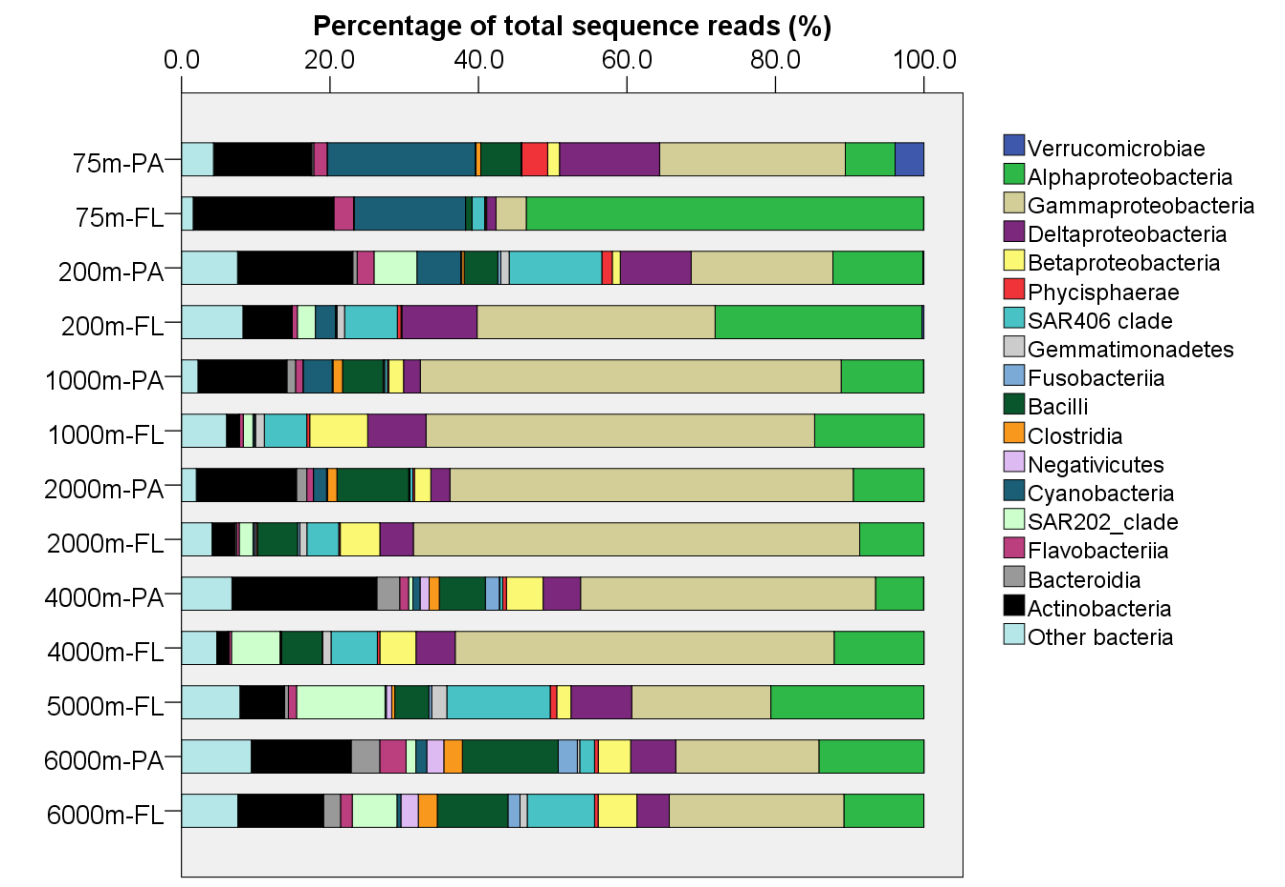


**Fig. S4.** The composition of bacterial communities at the class level. The analysis was based on the combined datasets from two original data sets, “Qiagen-338-806” and “MP-515-907”.

**Fig. S5** Variation of the Bray-Curtis similarities between the PA (filled circle) and FL (open circle) bacterial diversities with depth. Each symbol shows the similarity value between PA or FL bacterial community at deeper water depth and those of corresponding lifestyle at 75m.

## Supplementary Table

**Supplementary Table 1.** Bacterial taxa identified from the two individual datasets and the combined dataset.

|  | OTU | Species | Genus | Class | Phylum |
| --- | --- | --- | --- | --- | --- |
| MP-515-907 | 2438 | 1363 | 773 | 87 | 35 |
| Qiagen-338-806 | 1947 | 1037 | 596 | 85 | 34 |
| Combined | 3313 | 1663 | 905 | 98 | 41 |
